# Supplementary material for: Combined effects of temperature, photoperiod, and salinity on reproduction of the brine shrimp Artemia sinica (Crustacea: Anostraca)
Source: PeerJ. 2023 Sep 25;11:e15945. doi: 10.7717/peerj.15945 (PMC10538291; doi:10.7717/peerj.15945)
Supplement: Supplemental Information 1 [file peerj-11-15945-s001.docx]

Table S1. Results (*p* values) of three-way (temperature, light hours, and salinity) ANOVAs

|  | T | L | S | T×L | T×S | L×S | T×L×S |
| --- | --- | --- | --- | --- | --- | --- | --- |
| Lifespan (d) | 0.000 | 0.088 | 0.008 | 0.001 | 0.000 | 0.105 | 0.116 |
| Pre-reproductive period (d) | 0.000 | 0.000 | 0.000 | 0.000 | 0.000 | 0.000 | 0.000 |
| Reproductive period (d) | 0.000 | 0.689 | 0.000 | 0.000 | 0.000 | 0.001 | 0.045 |
| Post-reproductive period (d) | 0.000 | 0.871 | 0.020 | 0.060 | 0.010 | 0.000 | 0.593 |
| Reproductive interval (d) | 0.000 | 0.338 | 0.004 | 0.375 | 0.254 | 0.077 | 0.365 |
| Number of broods | 0.000 | 0.581 | 0.000 | 0.000 | 0.004 | 0.000 | 0.003 |
| Total offspring | 0.000 | 0.921 | 0.000 | 0.000 | 0.018 | 0.000 | 0.031 |
| Offspring per brood | 0.000 | 0.017 | 0.000 | 0.000 | 0.000 | 0.000 | 0.000 |
| Offspring per day | 0.000 | 0.306 | 0.000 | 0.000 | 0.036 | 0.000 | 0.073 |
| Offspring per reproductive day | 0.000 | 0.000 | 0.000 | 0.000 | 0.000 | 0.018 | 0.301 |
| % Oviparous broods | 0.000 | 0.000 | 0.000 | 0.000 | 0.001 | 0.000 | 0.142 |
| % Oviparous offspring | 0.000 | 0.000 | 0.000 | 0.000 | 0.002 | 0.000 | 0.250 |
