# Supplementary material for: Combined effects of temperature, photoperiod, and salinity on reproduction of the brine shrimp Artemia sinica (Crustacea: Anostraca)
Source: PeerJ. 2023 Sep 25;11:e15945. doi: 10.7717/peerj.15945 (PMC10538291; doi:10.7717/peerj.15945)
Supplement: Supplemental Information 2 — Rn: ratio of average sizes of ovoviviparous to oviparous broods in the nth brood. [file peerj-11-15945-s002.docx]

Table S2. Comparison of numbers of nauplii and resting eggs per brood of *Artemia sinica* (only data from ≥10 females are shown).

Rn: ratio of average sizes of ovoviviparous to oviparous broods in the nth brood.

| T | L | S | R1 | R2 | R3 | R4 | R5 | R6 | R7 | R8 | R9 | R10 | R11 | R12 | R13 |
| --- | --- | --- | --- | --- | --- | --- | --- | --- | --- | --- | --- | --- | --- | --- | --- |
| 6 | 6 | 50 | 0.89 | 0.95 |  |  |  |  |  |  |  |  |  |  |  |
|  |  | 100 | 0.97 | 1.34 | 1.11 | 1.15 |  |  |  |  |  |  |  |  |  |
|  | 12 | 50 | 1.03 | 0.94 | 1.03 | 0.97 |  |  |  |  |  |  |  |  |  |
|  |  | 100 | 1.03 | 1.24 | 0.69 |  |  |  |  |  |  |  |  |  |  |
|  | 18 | 50 | 0.92 | 0.90 | 1.06 | 1.12 | 1.21 |  |  |  |  |  |  |  |  |
|  |  | 100 | 1.23 | 1.80 |  |  |  |  |  |  |  |  |  |  |  |
| 25 | 6 | 50 | 0.85 | 1.00 | 1.00 | 0.99 | 0.91 | 1.01 | 1.13 | 0.79 |  |  |  |  |  |
|  |  | 100 | 1.02 | 0.77 | 0.82 | 0.99 | 1.76 | 0.84 |  |  |  |  |  |  |  |
|  |  | 150 | 0.90 | 0.98 | 0.91 | 0.67 | 0.82 | 0.84 | 0.92 | 0.70 | 0.72 |  |  |  |  |
|  | 12 | 50 | 1.13 | 0.94 | 0.73 | 0.68 | 0.89 | 1.31 | 0.99 | 1.06 | 0.85 | 1.03 |  |  |  |
|  |  | 100 | 0.92 | 0.98 | 0.94 | 1.04 | 0.99 | 0.95 | 1.17 | 1.01 | 1.17 | 0.85 | 0.79 |  |  |
|  |  | 150 | 1.02 | 0.84 | 1.10 | 1.15 | 0.70 | 1.37 |  |  |  |  |  |  |  |
|  | 18 | 50 | 0.64 | 0.79 | 0.82 | 0.92 | 0.92 | 1.03 | 0.84 | 0.91 | 1.33 | 1.05 | 0.73 | 0.80 | 1.04 |
|  |  | 100 | 0.74 | 0.80 | 1.03 | 0.77 | 0.98 | 0.85 | 1.01 | 0.94 | 1.29 | 0.94 |  |  |  |
|  |  | 150 | 0.95 | 0.90 | 0.98 | 1.00 | 1.09 | 1.47 | 0.97 | 0.88 |  |  |  |  |  |
| 30 | 6 | 50 | 1.17 | 1.16 | 1.22 | 0.98 | 0.89 |  |  |  |  |  |  |  |  |
|  |  | 100 | 0.90 | 0.91 | 0.85 | 1.02 | 0.94 | 1.03 |  |  |  |  |  |  |  |
|  | 12 | 50 | 0.94 | 1.22 | 1.16 | 0.89 |  |  |  |  |  |  |  |  |  |
|  |  | 100 | 0.91 | 1.18 | 0.86 |  |  |  |  |  |  |  |  |  |  |
|  | 18 | 50 | 1.05 | 0.85 | 0.78 | 0.90 | 1.10 | 0.91 |  |  |  |  |  |  |  |
|  |  | 100 | 1.31 | 0.95 | 1.18 | 1.29 | 1.26 | 1.04 | 1.30 | 1.30 |  |  |  |  |  |
| Mean | | | 0.98 | 0.99 | 0.98 | 0.98 | 0.98 | 1.11 | 1.05 | 1.00 | 1.16 | 0.91 | 0.76 | 0.80 | 1.04 |
