# Supplementary material for: Combined effects of temperature, photoperiod, and salinity on reproduction of the brine shrimp Artemia sinica (Crustacea: Anostraca)
Source: PeerJ. 2023 Sep 25;11:e15945. doi: 10.7717/peerj.15945 (PMC10538291; doi:10.7717/peerj.15945)
Supplement: Supplemental Information 3 — N = 56. Significance levels are p < 0.05 (*) or p < 0.01 (**). [file peerj-11-15945-s003.docx]

Table S3. Linear correlation (Pearson simple correlation coefficient *r*-value) between lifespan and reproductive parameters in the treatment of T25-L12-S100.

N=56. Significance levels are *p*<0.05 (*) or *p*<0.01 (**).

|  | Lifespan | Pre-reproductive period | Reproductive period | Post-reproductive period | Reproductive interval | Number of broods | Total offspring | Offspring per day | Offspring per reproductive day | Offspring per brood | % Oviparous broods |
| --- | --- | --- | --- | --- | --- | --- | --- | --- | --- | --- | --- |
| Pre-reproductive period | -0.066 | 1.000 |  |  |  |  |  |  |  |  |  |
| Reproductive period | 0.968^**^ | -0.168 | 1.000 |  |  |  |  |  |  |  |  |
| Post-reproductive period | 0.102 | -0.097 | -0.116 | 1.000 |  |  |  |  |  |  |  |
| Reproductive interval | 0.065 | 0.230 | -0.011 | 0.199 | 1.000 |  |  |  |  |  |  |
| Number of broods | 0.929^**^ | -0.195 | 0.965^**^ | -0.115 | -0.173 | 1.000 |  |  |  |  |  |
| Total offspring | 0.777^**^ | -0.240 | 0.802^**^ | -0.034 | -0.260 | 0.881^**^ | 1.000 |  |  |  |  |
| Offspring per day | 0.597^**^ | -0.286^*^ | 0.644^**^ | -0.090 | -0.392^**^ | 0.766^**^ | 0.951^**^ | 1.000 |  |  |  |
| Offspring per reproductive day | 0.379^**^ | -0.234 | 0.402^**^ | -0.001 | -0.479^**^ | 0.564^**^ | 0.830^**^ | 0.939^**^ | 1.000 |  |  |
| Offspring per brood | 0.515^**^ | -0.213 | 0.532^**^ | 0.006 | -0.319^*^ | 0.634^**^ | 0.891^**^ | 0.957^**^ | 0.955^**^ | 1.000 |  |
| % Oviparous broods | 0.171 | 0.177 | 0.115 | 0.136 | 0.096 | 0.066 | 0.024 | -0.068 | -0.101 | -0.045 | 1.000 |
| % Oviparous offspring | 0.152 | 0.189 | 0.120 | 0.028 | 0.062 | 0.076 | 0.011 | -0.061 | -0.100 | -0.052 | 0.949^**^ |
