# Supplementary material for: Combined effects of temperature, photoperiod, and salinity on reproduction of the brine shrimp Artemia sinica (Crustacea: Anostraca)
Source: PeerJ. 2023 Sep 25;11:e15945. doi: 10.7717/peerj.15945 (PMC10538291; doi:10.7717/peerj.15945)
Supplement: Supplemental Information 4 — Here the “optimal conditions” are defined as: temperature, about 25 °C (often used as the standard temperature for Artemia culture); photoperiod, L12: D12 (close to natural daylight) or L24: D0 (if only continuous light was adopted in references); salinity, the salinity at which the best reproductive parameters (criterion: A. shortest pre-reproductive period; B. maximum offspring per day in reproductive period) were recorded. [file peerj-11-15945-s004.docx]

Table S4. Comparison of reproductive parameters recorded for some *Artemia* species or populations under “optimal conditions”.

Here the “optimal conditions” are defined as: temperature, about 25°C (often used as the standard temperature for *Artemia* culture); photoperiod, L12: D12 (close to natural daylight) or L24: D0 (if only continuous light was adopted in references); salinity, the salinity at which the best reproductive parameters (criterion: A. shortest pre-reproductive period; B. maximum offspring per day in reproductive period) were recorded.

| Species | Population | Criterion | T (℃) | L (h) | S (PSU) | Pre-reproductive period (d) | Offspring per female | Offspring per brood | Offspring per reproductive day per female | oviparity % | References |
| --- | --- | --- | --- | --- | --- | --- | --- | --- | --- | --- | --- |
| *Artemia sinica* | Yuncheng Salt Lake, China | A | 24 | / | 120 | 22.8 | 287.4 | 47.0 | 10.4 | 28.5 | Browne & Wanigasekera, 2000 |
|  |  | B | 24 | / | 180 | 26.0 | 121.0 | 40.9 | 18.6 | 80.3 |  |
|  |  | **A** | **25** | **12** | **100** | **19.7** | **515.1** | **56.9** | **12.2** | **21.3** | **Present study** |
|  |  | **B** | **25** | **12** | **50** | **26.6** | **532.9** | **65.9** | **16.2** | **41.6** |  |
| *Artemia franciscana* | San Francisco Bay, USA | / | 24 | 24 | 90 | 30.5 | 1619 | 111.4 | 27.8 | 28.1 | Browne et al., 1984 |
|  |  | A; B | 24 | 24 | 90 | 30.5 | 1619 | 111.4 | 27.8 | 28.1 | Browne et al.,1988 |
|  |  | A | 25 | 12 | 100 | 20.3 | 1348.0 | 86.8 | 23.0 | 15.6 | Triantaphyllidis et al., 1995 |
|  |  | B | 25 | 12 | 35 | 22.9 | 882.3 | 89.6 | 32.0 | 49.9 |  |
|  |  | A | 24 | / | 120 | 24.2 | 470.5 | 74.6 | 27.5 | 22.1 | Browne & Wanigasekera, 2000 |
|  |  | B | 24 | / | 60 | 35.0 | 66.0 | 44.8 | 28.2 | 0.0 | Browne & Wanigasekera, 2000 |
|  | Lake Chaplain, USA | / | 24 | 24 | 90 | 31.6 | 1057 | 95.6 | 23.0 | 18.4 | Browne et al.,1984 |
|  | Cabo Rojo, USA | / | 24 | 24 | 90 | 28.9 | 969 | 78.0 | 20.1 | 29.3 | Browne et al.,1984 |
| *Artemia persimilis* | Hidalgo, Argentina | / | 24 | 24 | 90 | 35.1 | 293 | 80.4 | 31.6 | 44.1 | Browne et al.,1984 |
|  |  | A; B | 24 | / | 120 | 19.4 | 426.5 | 61.6 | 17.5 | 91.9 | Browne & Wanigasekera, 2000 |
| *Artemia salina* | Chott Ariana, Tunisia | / | 24 | 24 | 90 | 32.5 | 159 | 20.7 | 4.7 | 89.3 | Browne et al.,1984 |
|  | Lake Larnaca, Cyprus | / | 24 | 24 | 90 | 41.2 | 112 | 29.8 | 10.4 | 100.0 | Browne et al.,1984 |
|  |  | A | 24 | 24 | 90 | 45.1 | 74 | 23.5 | 7.9 | 100.0 | Browne et al.,1988 |
|  |  | A; B | 24 | / | 120 | 20.3 | 130.7 | 35.6 | 8.5 | 100.0 | Browne & Wanigasekera, 2000 |
|  | Megrine, Tunisia | A; B | 24 | 24 | 90 | 46.6 | 105 | 23.4 | 9.4 | 99.0 | Browne et al.,1988 |
|  | Sebkha of Sidi El Hani, Tunisia | / | 25 | 16 | 90 |  | 527.3 | 60.7 |  | 93.1 | Sellami et al., 2021 |
| *Artemia urmiana* | Lake Urmia, Iran | A | 25 | / | 180 | 31.0 | 416.7 | 34.2 | 6.9 | 80.7 | Abatzopoulos et al., 2006 |
|  |  | B | 25 | / | 100 | 31.2 | 341.1 | 43.2 | 13.8 | 58.2 |  |
|  |  | A | 27 | / | 75 | 21.5 | 194.3 | 47.5 |  | 43.6 | Agh et al., 2008 |
|  |  | A; B | 24 | 14 | 100 | 19.2 | 935.2 | 17.1 | 19.0 | 22.7 | Asil et al., 2013 |
| Parthenogenetic *Artemia* | Cadiz, Spain | / | 24 | 24 | 90 | 43.4 | 645 | 52.4 | 10.5 | 49.1 | Browne et al.,1984 |
|  |  | B | 24 | 24 | 90 | 46.6 | 689 | 60.0 | 11.1 | 28.4 | Browne et al.,1988 |
|  | Giraud, France | / | 24 | 24 | 90 | 40.2 | 566 | 74.8 | 16.5 | 10.0 | Browne et al.,1984 |
|  |  | A; B | 24 | 24 | 90 | 26.6 | 596 | 65.6 | 13.2 | 29.9 | Browne et al.,1988 |
|  | Izmir, Turkey | / | 24 | 24 | 90 | 40.3 | 1160 | 80.7 | 13.7 | 15.2 | Browne et al.,1984 |
|  | Madras, India | / | 24 | 24 | 90 | 39.8 | 1442 | 108.4 | 21.6 | 13.8 | Browne et al.,1984 |
|  | Kutch, India | / | 24 | 24 | 90 | 30.8 | 1531 | 78.2 | 14.1 | 3.0 | Browne et al.,1984 |
|  |  | A; B | 24 | 24 | 90 | 24.8 | 818 | 62.8 | 12.3 | 17.0 | Browne et al.,1988 |
|  | Tanggu, China | A | 25 | 12 | 100 | 20.6 | 331.2 | 66.2 | 18.0 | 7.8 | Triantaphyllidis et al., 1995 |
|  |  | B | 25 | 12 | 60 | 20.7 | 186.0 | 67.0 | 23.0 | 13.0 |  |
|  | Margherita di Savoia, Italy | A | 24 | / | 180 | 36.4 | 541.7 | 42.7 | 7.6 | 81.7 | Browne & Wanigasekera, 2000 |
|  |  | B | 24 | / | 120 | 40.0 | 603.0 | 57.3 | 9.8 | 17.1 |  |
|  | Thessaloniki, Greece | A | 22 | / | 50 | 32.0 | 180.6 | 36.5 |  | 14.6 | Abatzopoulos et al..,2003 |
|  |  | B | 22 | / | 80 | 33.1 | 482.0 | 93.3 |  | 71.2 |  |
|  | Bohai Bay, China | A | 25 | / | 70 | 11.2 |  |  |  | 6.5 | Sui et al., 2013 |
|  | Gaav Khooni Isfahan, Iran | A | 27 | / | 120 | 8.4 |  |  |  |  | Aalamifar et al., 2014 |
|  | Around Lake Urmia, Iran | A | 27 | / | 80 | 27.4 |  |  |  |  | Aalamifar et al., 2014 |

**References**

**Aalamifar H, Agh N, Malekzadeh R, Aalinezhad M. 2014.** A comparative study on the effect of different salinities on the survival, growth, life span and morphometric characteristics cyst of two parthenogenetic species of *Artemia* (Gaav Khooni wetlands of Isfahan, ponds around Lake Urmia) from Iran. *Asian Journal of Biological Sciences* 7(6): 242-251.

**Abatzopoulos TJ, Baxevanis AD, Triantaphyllidis GV, Criel G, Pador EL, Stappen GV, Sorgeloos P. 2006.** Quality evaluation of Artemia urmiana Günther (Urmia Lake, Iran) with special emphasis on its particular cyst characteristics (International Study on Artemia LXIX). Aquaculture 254(1-4): 442-454.

**Abatzopoulos TJ, El-Bermawi N, Vasdekis C, Baxevanis AD, Sorgeloos P. 2003.** Effects of salinity and temperature on reproductive and life span characteristics of clonal *Artemia* (International Study on *Artemia*. LXVI). *Hydrobiologia* 492(1-3): 191-199.

**Agh N, Van Stappen G, Bossier P, Sepehri H, Lotfi V, Rouhani S M, Sorgeloos P. 2008.** Effects of salinity on survival, growth, reproductive and life span characteristics of *Artemia* populations from Urmia Lake and neighboring lagoons. *Pakistan Journal of Biological Sciences* 11: 164-172.

**Asil SM, Fereidouni AE, Ouraji H, Khalili KJ. 2013.** Effects of different light intensities on growth, survival, reproductive and life span characteristics of *Artemia urmiana* (Günther 1890). *Aquaculture Research* 44(4): 554-566.

**Browne RA, Davis LE, Sallee SE. 1988.** Effects of temperature and relative fitness of sexual and asexual brine shrimp *Artemia*. *Journal of Experimental Marine Biology and Ecology* 124(1): 1-20.

**Browne RA, Sallee SE, Grosch DS, Segreti WO, Purser SM. 1984.** Partitioning genetic and environmental components of reproduction and lifespan in *Artemia*. *Ecology*, 65(3): 949-960.

**Browne RA, Wanigasekera G. 2000.** Combined effects of salinity and temperature on survival and reproduction of five species of *Artemia*. *Journal of Experimental Marine Biology and Ecology* 244(1):29-44.

**Sellami I, Naceur HB, Kacem A. 2021.** Reproductive performance in successive generations of the brine shrimp *Artemia salina* (Crustacea: Anostraca) from the Sebkha of Sidi El Hani (Tunisia). *Animal Reproduction Science* 225(5847):106692.

**Sui LY, Wang J, He H, Deng YG. 2013.** Effect of salinity on survival and reproductive performance of different *Artemia* strains. *Marine Sciences* 37(11): 41-47. (in Chinese with English abstract)

**Triantaphyllidis GV, Poulopoulou K, Abatzopoulos TJ, Perez CA, Sorgeloos P. 1995.** International study on *Artemia* XLIX. Salinity effect on survival, maturity, growth, biometrics, reproductive and life span characteristics of a bisexual and a parthenogenetic population of *Artemia*. *Hydrobiologia* 302 (3): 215-227.
